# Percutaneous Cerebral Angioplasty for Refractory Middle Cerebral Artery Stenosis Due to Varicella-Zoster Virus-Related Vasculopathy: A Case Report

**DOI:** 10.7759/cureus.69773

**Published:** 2024-09-20

**Authors:** Soichiro Matsubara, Makoto Nakajima, Yasuyuki Kaku, Akitake Mukasa, Mitsuharu Ueda

**Affiliations:** 1 Department of Neurology, Graduate School of Medical Sciences, Kumamoto University, Kumamoto, JPN; 2 Department of Neurosurgery, Kumamoto University Hospital, Kumamoto, JPN

**Keywords:** angioplasty, endovascular treatment, stroke, varicella-zoster, vasculopathy

## Abstract

An 81-year-old man was admitted to our hospital with left hemiplegia after treatment for herpes zoster of the first branch of the right trigeminal nerve. CSF examination revealed an elevated varicella-zoster virus (VZV) antibody index. Brain MRI showed cerebral infarction in the right middle cerebral artery (MCA) territory and vessel wall thickening and enhancing effects at the ipsilateral MCA. Despite the standard treatment, the MCA stenosis progressed with recurrent infarcts. Percutaneous cerebral angioplasty was performed to the distal portion of the right MCA without deterioration. This case can provide a treatment option for refractory progressive VZV vasculopathy.

## Introduction

Varicella-zoster virus (VZV) vasculopathy, which involves both large and small arteries, is an infrequent cause of acute stroke. The characteristic pathology of VZV vasculopathy matches that of granulomatous arteritis, which occurs even in immunocompetent individuals [1-5]. The branches of the internal carotid artery are frequently affected, especially the middle cerebral artery (MCA) [1-3]; vascular abnormalities are reported in approximately 70% of patients with stroke secondary to VZV infection [3]. Antiviral drugs and immunotherapy are used in addition to antithrombotic agents; there are miserable cases of progressive vascular stenosis [1-5]. Percutaneous transluminal angioplasty is sometimes indicated in the treatment of refractory cases of stroke with cerebral arterial stenosis due to atherosclerosis [6,7]. However, there are limited reports on intracranial vasculitis, like VZV vasculopathy. Here, we report a case of refractory VZV vasculopathy and discuss its treatment.

## Case presentation

**Figure 1:**
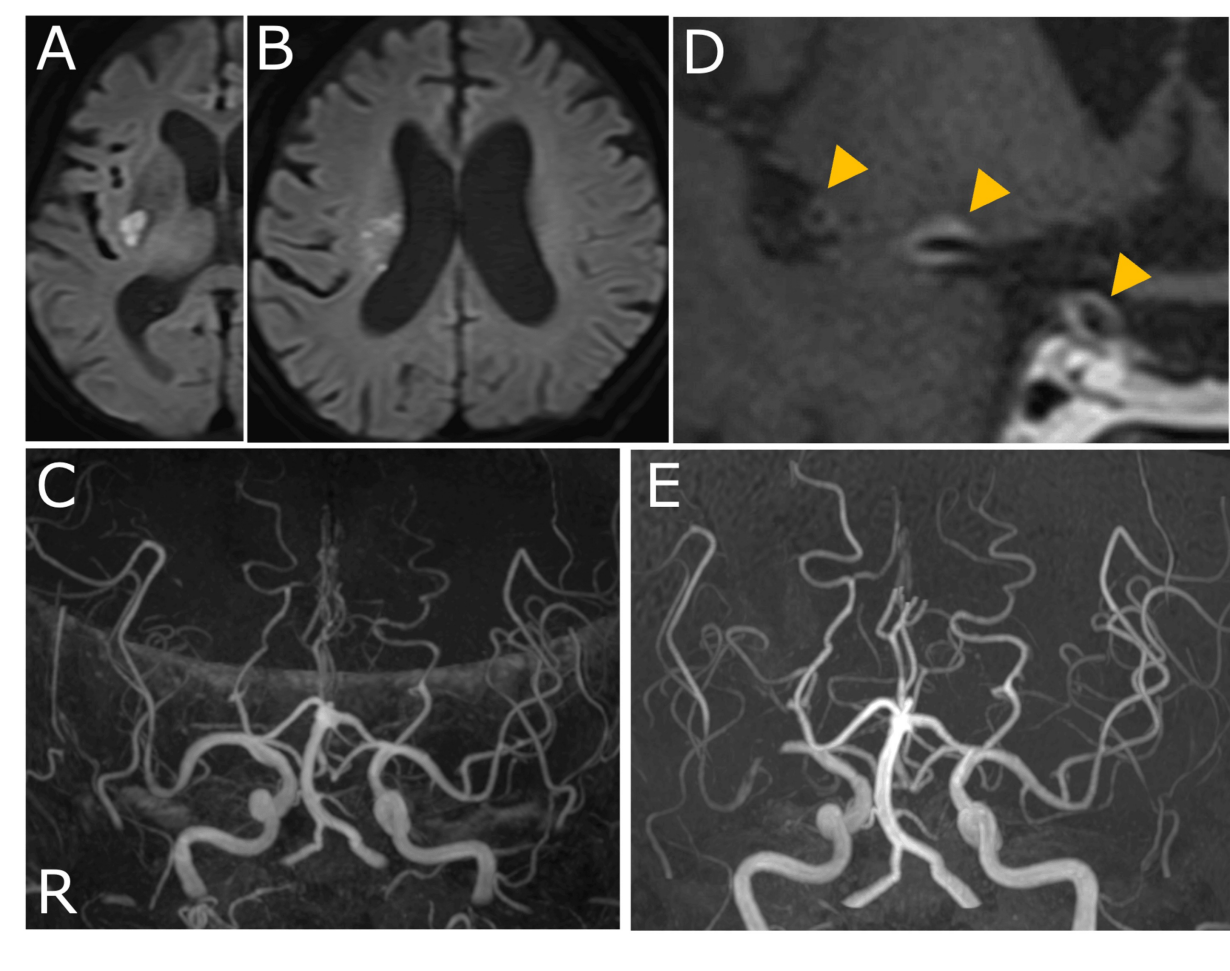
Brain MRI at initial assessment and disease progression. (A, B) Diffusion-weighted images on admission show a small region with diffusion restriction in the right putamen and blurred and small patchy lesions in the corona radiata on Day 45. (C) MRA showed no cerebral artery stenosis on Day 45. (D) A contrast-enhanced T1-weighted image reveals circumferential vessel wall thickening and gadolinium enhancement at the right ICA terminus and the distal portion of the right middle cerebral artery (MCA) (arrowheads) on Day 45. (E) MRA showed right severe MCA stenosis and reduced distal vessel intensity on Day 82.

An 81-year-old man with no significant medical history presented with herpes zoster cutaneous lesions in the ophthalmic division of the right trigeminal nerve (V1, Day 0). Oral acyclovir was initiated by a primary care physician. On Day 20, the pain worsened, and he was admitted to the anesthesiology department of the previous hospital, where nerve blocks and other treatments were performed. He then developed left hemiplegia and dysarthria and was transferred to our hospital on Day 45. Examination showed superficial hypoesthesia and allodynia in the same face area (V1) and mild left hemiplegia, including facial paralysis; the NIH Stroke Scale score was 3. Brain MRI diffusion-weighted imaging (DWI) showed subacute cerebral infarction in the right basal ganglia (Figures 1A, 1B); furthermore, hyperintense signal changes were observed on DWI and fluid-attenuated inversion recovery (FLAIR) along the right spinal trigeminal nucleus. There was no stenosis of the middle cerebral artery (MCA) on MR angiography (MRA) (Figure 1C); however, T1-weighted contrast-enhanced vessel wall images showed vessel wall thickening and gadolinium enhancement at the right internal carotid artery (ICA) terminus and the distal portion of the right MCA (Figure 1D). Blood test results were unremarkable, including coagulation and rheumatologic testing (Table 1). Cerebrospinal fluid (CSF) cell count was 13/μL (100% mononuclear cells); CSF protein was elevated at 82 mg/dL (Table 2). Although PCR for VZV DNA was negative in CSF, VZV vasculopathy was diagnosed based on the elevated antibody index value of the VZV of 7.97.

**Table 1:** The laboratory parameters along with the normal range for reference. WBC: white blood cell, RBC: red blood cell, Hb: hemoglobin, PLT: platelet, T-Bil: total bilirubin, AST: aspartate aminotransferase, ALT: alanine aminotransferase, LDH: amylase, CK: creatine kinase, γ-GTP: γ-glutamyl transpeptidase, BUN: blood urea nitrogen, Cre: creatinine, Glu: glucose, HbA1c: hemoglobin A1c, HDL: high-density lipoprotein, LDL: low-density lipoprotein, BNP: brain natriuretic peptide, F-T4: free thyroxine, sIL-2R: soluble interleukin-2 receptor, RPR: rapid plasma regain, TPLA: Treponema pallidum latex agglutination, HSV: herpes simplex virus, VZV: varicella-zoster virus, CMV: cytomegalovirus, PR3-ANCA: proteinase-3-anti-neutrophil cytoplasmic antibodies, MPO-ANCA: myeloperoxidase-anti-neutrophil cytoplasmic antibodies.

Parameters	Values	Reference range
WBC (×10^3/μL)	7.20	3.3-8.6
RBC (×10^6/μL)	4.10	4.35-5.55
Hb (g/dL)	12.7	13.7-16.8
PLT (×10^3/μL)	22.6	158-348
T-Bil (mg/dL)	1.1	0.4-1.5
AST (U/L)	29	13-30
ALT (U/L)	31	10-42
LDH (IFCC) (U/L)	210	124-222
CK (U/L)	57	59-248
γ-GTP (U/L)	29	13-64
Na (mmol/L)	135	138-145
K (mmol/L)	4.6	3.6-4.8
Cl (mmol/L)	100	101-108
BUN (mg/dL)	20.7	8.0-20.0
Cre (mg/dL)	0.93	0.65-1.07
HbA1c（％）	6.0	4.9-6.0
Glucose (mg/dL)	112	73-109
HDL-Chol (mg/dL)	44	38-90
LDL-Chol (mg/dL)	107	65-163
BNP (pg/mL)	15.7	<18.4
F-T4 (ng/dL )	1.11	1.1-1.8
TSH (μIU/mL )	0.66	0.5-5.0
sIL-2R (U/mL)	464	121-613
RPR test	Negative	
TPLA test	Negative	
HSV IgG antibody (EIA)	>128	>3.9
HSV IgM antibody (EIA)	0.35	>1.2
VZV IgG antibody (EIA)	100.7	>3.9
VZV IgM antibody (EIA)	0.40	>1.20
CMV IgG antibody (EIA)	243.1	>6.0
CMV IgM antibody (EIA)	<0.85	>1.0
Antinuclear antibody	Negative	
Rheumatoid factor	Negative	
PR3-ANCA	Negative	
MPO-ANCA	Negative	

**Table 2:** The cerebrospinal fluid analysis along with the normal range for reference. The results of the cerebrospinal fluid tests are shown. VZV: varicella-zoster virus.

Parameters	Values	Reference range
Total protein (mg/dL)	82	8-43
Glucose (mg/dL)	52	50-75
IgG (mg/dL)	9.35	
IgG index	0.71	
Cell count (/μL)	13	<5
Lymphocyte	100%	
VZV IgG antibody (EIA)	7.61	
VZV IgM antibody (EIA)	0.32	
VZV antibody index	7.97	<2
VZV-DNA PCR	negative	

**Figure 2:**
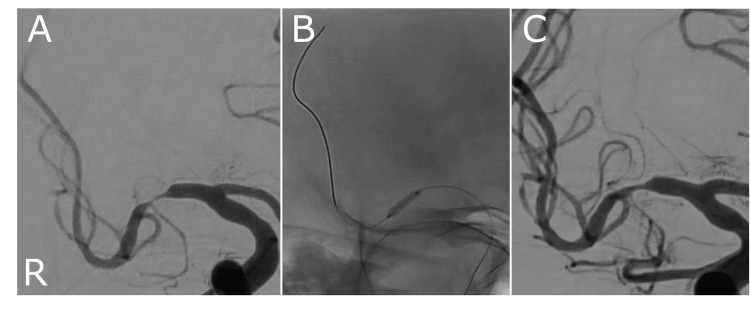
Angiogram of the right middle cerebral artery during the angioplasty. (A) The angiogram of the right middle cerebral artery (MCA) before endovascular therapy demonstrates severe stenosis in the right MCA M1 portion with distal contrast delay. (B) Inflation of the balloon catheter (Unryu XP 2.0 mm × 10 mm, Kaneka Medix, Osaka, Japan). (C) Dilation is visible after single angioplasty, with improved distal perfusion and no intraoperative restenosis.

After the admission, administration of aspirin (200 mg), clopidogrel (75 mg), intravenous acyclovir (10 mg/kg, q8h, 14 days), and short-term intravenous methylprednisolone (1,000 mg/day, three days), cerebral infarctions recurred in the corona radiata and basal ganglia. Acyclovir was changed to oral valacyclovir (3,000 mg/day) and continued until discharge. In addition to the progression of left hemiplegia and dysarthria, mild left hemispatial neglect also appeared. Magnetic resonance angiography revealed gradual progression of right MCA stenosis without cortical infarction. On Day 83, the patient was re-treated with steroids; however, further progression of the stenosis was observed. Severe right MCA M1 segment stenosis and a distal blood flow delay were observed on cerebral angiography (Figure 2A). The patient was refractory to medical treatment, and percutaneous cerebral angioplasty using a balloon catheter was performed on Day 90 (Figure 2B). The treatment was completed without complications, including intraoperative restenosis. Distal blood flow in the right MCA also improved (Figure 2C). The symptoms remained stable, without signs of worsening left hemispatial neglect, and the peripheral MCA branches were well-delineated on MR angiography on Days 91, 97, and 104. On Day 105, the patient was transferred to a rehabilitation hospital. Acyclovir was discontinued one month after transfer, and dual antiplatelet therapy was continued at the following institution. Brain MRI was performed nine months after onset, and no recurrent stroke, restenosis, or aneurysm formation was observed.

## Discussion

VZV vasculopathy can damage both large and small vessels, and stenoses or occlusions were previously observed in 70% of patients [3]. Multiple stenoses can occur, which are observed as gadolinium enhancement in the walls of major vessels. VZV vasculopathy seems to be associated with productive viral infections in the arteries, as evidenced by the presence of multinucleated giant cells, VZV DNA, and VZV antigens [1]. Ischemic stroke is the most common type of stroke in VZV vasculopathy; however, the disruption of the media layer contributes to aneurysm formation, and cerebral or subarachnoid hemorrhages may also occur [1-5].

The present case requires careful consideration of whether there was an intracranial infection with VZV. VZV meningitis can often be asymptomatic in elderly patients. In addition to this point, whether the present cerebral infarction was caused solely by VZV vasculopathy should be thoroughly discussed. The patient was elderly, and atherosclerotic changes may have potentially overlapped with the ischemic lesions of the brain parenchyma and the progressive arteriosclerosis. It would be better if the virus could be proved by direct biopsy of the vascular lesion, but this is often difficult in clinical practice, including in this case. However, the findings of vasculitis in MCA, in this case, were (1) concentric enhancing effects rather than eccentric enhancing effects on contrast-enhanced MRI and (2) patchy multiple infarcts in the corona radiata and basal ganglia, which were presumed to be findings that VZV vasculopathy was more important effect to progressive clinical course rather than typical atherosclerosis [1-3,6]. In addition to these radiological features, in virological studies, the VZV-PCR of CSF was negative. Still, PCR is known to be less sensitive in VZV vasculopathy, and the VZV antibody index is used to examine the association. This index was higher than the reference value (>2), supporting an association [1-3]. The risk of cerebral infarction after VZV infection increases two weeks after infection, which is consistent with the timing of the onset of the syndrome [2].

In addition to the standard treatment, including antithrombotic agents for ischemic stroke, short-term corticosteroids and continuous acyclovir administration are recommended for VZV vasculopathy. The prognosis of this disease is unfavorable in many cases because a wide range of intracranial vessels, from small to large, are affected [1-3]. As in previous reports, progressive cerebral artery stenosis was observed in this patient and was refractory to these medical treatments. Thus, further treatment was clinically necessary to prevent further symptom progression, such as hemispatial neglect.

Angioplasty is a promising treatment option for cerebral infarction with progressive cerebral artery stenosis due to atherosclerosis; however, the indications for treatment are still controversial because vessel fragility due to vasculopathy can lead to severe complications, including dissection or subarachnoid hemorrhage [7,8]. Transluminal vasodilation efficacy and safety as an adjunctive intervention are more carefully determined in patients with infectious intracranial vasculopathy. This therapy has also been used rarely in some patients with parainfectious cerebral vasospasm with subarachnoid hemorrhage [5]. Therefore, this intervention is not ruled out, even in the case of a hemorrhagic event. To further consider the indications for treatment in the present case, the efficacy of the treatment regarding vasculitis was further examined due to other etiology treated with similar vessel sizes. Previous reports have shown good outcomes in patients with major arterial stenosis resulting from vasculitis, where the targeted vessel size was comparable with that of the present case [9-12]. Favorable outcomes have also been reported in angioplasty performed in vertebral artery stenosis caused by giant cell vasculitis [9-11] or in middle cerebral artery stenosis caused by neurosarcoidosis [12]. Local administration of vasodilators and stenting can also be considered to treat stenotic lesions. In this case, vasodilators were not administered to treat vasospasm because the patient had persistent vascular stenosis and contrast-enhanced MRI suggested an inflammatory pathology. No additional stenting was performed as no restenosis was observed after a single angioplasty. In our case, we carefully considered the indications for treatment, and the patient could avoid worsening neurological disability. Therefore, angioplasty should not be excluded as a treatment for vasculitis-induced intracranial stenosis.

## Conclusions

In this case, we experienced a refractory intracranial cerebral artery secondary to VZV infection. We carefully considered the indications for percutaneous transluminal angioplasty, and additional intervention could prevent poor outcomes. Endovascular therapy could be an option for treating refractory intracranial cerebral arterial stenosis due to VZV vasculopathy.
